# Nano-Lipids Based on Ginger Oil and Lecithin as a Potential Drug Delivery System

**DOI:** 10.3390/pharmaceutics14081654

**Published:** 2022-08-09

**Authors:** Hung Quach, Tuong-Vi Le, Thanh-Thuy Nguyen, Phuong Nguyen, Cuu Khoa Nguyen, Le Hang Dang

**Affiliations:** 1 Institute of Applied Materials Science, Vietnam Academy of Science and Technology, Ho Chi Minh City 700000, Vietnam; 2 Vietnam Academy of Science and Technology, Graduate University of Science and Technology, Ho Chi Minh City 700000, Vietnam; 3 Faculty of Chemical Technology, HCMC University of Food Industry, Ho Chi Minh City 700000, Vietnam

**Keywords:** lecithin, ginger oil, essential oil, nano-lipid, drug delivery system

## Abstract

Lipid nanoparticles based on lecithin are an interesting part of drug delivery systems. However, the stability of lecithin nano-lipids is problematic due to the degradation of lecithin, causing a decrease in pH. In this study, the modification of the conventional nano-lipid-based soybean lecithin was demonstrated. Ginger-oil-derived Zingiber officinale was used along with lecithin, cholesterol and span 80 to fabricate nano-lipids (GL nano-lipids) using a thin-film method. TEM and a confocal microscope were used to elucidate GL nano-lipids’ liposome-like morphology. The average size of the resultant nano-lipid was 249.1 nm with monodistribution (PDI = 0.021). The ζ potential of GL nano-lipids was negative, similarly to as-prepared nano-lipid-based lecithin. GL nano-lipid were highly stable over 60 days of storage at room temperature in terms of size and ζ potential. A shift in pH value from alkaline to acid was detected in lecithin nano-lipids, while with the incorporation of ginger oil, the pH value of nano-lipid dispersion was around 7.0. Furthermore, due to the richness of shogaol-6 and other active compounds in ginger oil, the GL nano-lipid was endowed with intrinsic antibacterial activity. In addition, the sulforhodamine B (SRB) assay and live/dead imaging revealed the excellent biocompatibility of GL nano-lipids. Notably, GL nano-lipids were capable of carrying hydrophobic compounds such as curcumin and performed a pH-dependent release profile. A subsequent characterization showed their suitable potential for drug delivery systems.

## 1. Introduction

Lipid-based nano-carriers have been considered a promising tool for drug delivery. Because they resemble the natural biological structure of cell membranes and vesicles, lipid nano-carriers are able to enter cells via membrane fusion without compromising the intrinsic function of the carrier [1,2,3,4]. Lipid-based nano-carriers have two parts: the lipid matrix and an aqueous phase. This structure provides excellent advantages in packing both hydrophobic and hydrophilic agents [1,3]. In addition, formation of the lipid carrier is totally dependent on the physical interaction of lipid components through controlling the organic and aqueous phases, allowing self-assembly into the defined structure [5]. Therefore, lipid carrier production can be scaled up easily by using various suitable large-scale production techniques [6].

The basic components of lipid carriers are lipids, surface stabilizers and/or lipid excipients [5,6,7]. Lipids play a critical in the formation of the carrier’s shape [7]. In fact, amphiphilic lipids such as phospholipid are the common choice for production because they can self-assemble into micelles in an aqueous environment and mimic the lipid bilayer of cell membranes [7,8]. Currently, edible lipids with rich sources of phospholipids have generated much interest [4]. Lecithin is one of the most common natural edible lipids [9] and has been recognized as safe by the Food and Drug Administration [10]. Lecithin is a yellow-brown fatty substance which can be found in both animals and plants [7]. Among the different types of lecithin, soy lecithin has attracted a lot of interest in drug delivery system research due to the abundance sources as well as the relatively low production cost [8]. Further, soy lecithin contains a mixture of phospholipid compounds that is similar to the lipid bilayer cell membranes [9,10]. Unlike lecithin from animal or synthetic sources, along with 60–75% phospholipids, triglycerides and unsaturated fatty acid (linoleic, oleic, palmitic and α-linolenic acids) can be found in soy lecithin [11]. This composition is advantageous for application in the cardiac system, compared with their source of lecithin [11]. Various nano-lipid-based soy lecithins have been introduced and have shown good performance in drug delivery [9,12]. However, there is concern about the long-term physicochemical stability of lecithin nano-carriers [13,14,15]. When it degrades, lecithin can produce an acidic environment, a phenomenon that could represent a problem for parenteral administration [15].

Research has indicated that essential oils (EEs) can also be included in lipid formulations [3,16,17,18,19]. Together with monoterpenes and phenylpropenes, EEs are enriched in essential fatty acids, and could be an alternative carrier that avoids the potential problems of lecithin. A lipid carrier prepared with the EE of Cymbopogon flexuosus (lemongrass) offers greater stability at an ambient temperature [16]. In addition, the combination of EEs could help increase the amount of encapsulated hydrophobic drug or vitamin, allowing the sustainable release of the cargo and enhancing the cell permeation [3]. Meghea et al. [17] found that lipid nano-carriers prepared with linseed oil showed greater encapsulation of the drug and a gradual slow release compared with a conventional lipid carrier. Kumar et al. [18] produced a lipid carrier with the addition of linseed oil and found the improved permeability parameters and therapeutic value of thiocolchicoside. The use of palm oil in the lecithin–Tween 80–glycerol formulation developed by Arbain et al. [19] encapsulated a large amount of quercetin and was efficient for aerosol delivery. Another important factor to consider in the development of lipid carriers with EEs is the synergy between the EE and the loading agent [3]. Ginger oil [20], frankincense oil [21], garlic oil [22] and pomegranate seed oil [23], among others, display various medicinal properties, such as antimicrobial, antioxidant, anticarcinogen and sedative activity. Thus, EEs represent an excellent resource for fabricating functional lipid nano-carriers. 

In this work, conventional lecithin nano-lipids were modified with ginger oil. This substance has long been used as an alternative medicine and has been certified as a safe agent for food by the FDA [17]. Due to its lipophilicity, ginger oil could be located inside the lipid layer of lecithin nano-lipids. Ginger oil includes polyphenols (shogaol, gingerol, andparadols...), alkaloids, saponins, tannins, glycosides, flavonoids and glycolipids and fatty acids, all of which have therapeutic value [24,25]. Notably, ginger oil can prevent free-radical-induced damage to macromolecules [26]. Therefore, the addition of ginger oil in conventional lecithin nano-lipids could produce an effective drug carrier. In this study, ginger oil was extracted from Vietnam ginger root (Zingiber officinale) and then characterized by gas chromatography–mass spectrometry (GC-MS) and high-performance liquid chromatography (HPLC) before being used in the lipid fabrication process. The resultant GL nano-lipid batches were examined in terms of size, zeta potential and stability over 60 days of storage at room temperature. To examine whether GL nano-lipids could be potential drug carriers, their cytotoxic profile and ability to carry hydrophobic bioactive molecules (curcumin) were determined. This study has important implications for expanding current concepts of drug delivery with a multifunctional lipid nano-carrier based on EEs.

## 2. Results and Discussion

### 2.1. Characterization of Ginger Oil

**Figure 1:**
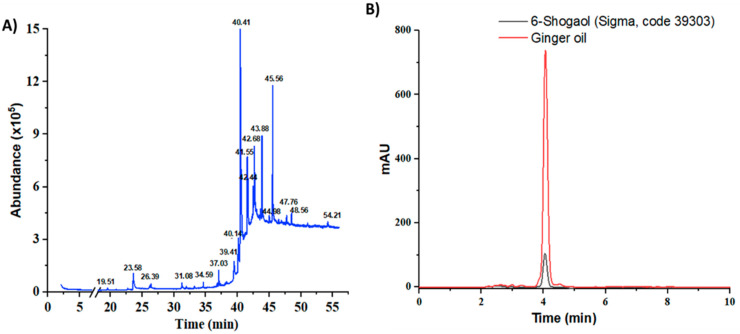
GC-MS chromatogram of edible ginger oil (**A**) and HPLC chromatograms of the representative ginger oil extracts in compared to standard 6-shogaol (**B**).

Based on the raw weight, Z. *officinale* yielded 0.7%. The chemical volatile composition of the resultant ginger oil was identified through gas chromatography—mass spectrometry (GC-MS) analysis. *Five* bioactive compounds were found in the obtained ginger oil within 55 min of retention time (Figure 1A, Table S1 and Figure S1). Most of these compounds have a boiling point above 80 °C. The identified compounds included fatty acid ester, geraniol, gingerdiol, shogaols and gingerols, similarly to what has been reported in the literature [24,25]. The compound with a retention time of 40.41 min was the most abundant (63.13%). This compound had [M]*^+^* peaks at *m*/*z* 276, a base peak at *m*/*z* 137(4-hydroxy-3-methoxybenzyl cation, [M*^+^*H-C*_13_*H*_22_*O]*^+^*) and some mass fractions such as m/z 205 (*the cleavage of the C-5*/*C-6 bond in the dehydrated ions at m*/*z = 276*) and *m*/*z* 119 (due to the loss of methyl group from *m*/*z* 137 fragment ion); this profile is characteristic of 6-shogaol (Figure S2) [26]. The component with a retention time of 41.55 was confirmed as [8]-Shogaol based on its mass spectrum (Figure S3) ([M]+ peak at *m*/*z* 304, a base peak at *m*/*z* 137 and a strong peak at *m*/*z* 205). This compound accounted for 6.29% of total volatile compounds. The last compound had a retention time of 43.88 min (7.73% of the volatile compounds). The mass spectrum showed [M]+ peaks at *m*/*z* 430 along with a base peak at *m*/*z* 137 (Figure S4). In addition, this spectrum had peaks at *m*/*z* 278, 279 in the mid-mass region, peaks at *m*/*z* 131, 138, 151 and 163 in the low-mass region and *a peak* at *m*/*z* 69, suggesting that this compound was [6]-Gingerdiol (2E)-geranial acetal. The extract also contained geraniol, a new class of chemoprevention agent [27]. It represented 1.95% of the total volatile compounds (retention time of 23.57, mass spectrum showing a peak at *m*/*z* 154 and a base peak at *m*/*z* 69, (Figure S5)).

The goal of this extraction was to obtain a 6-shogaol-rich ginger extract. Therefore, the extraction process was performed with two solvent-phase extractions. First, ethanol and heat were used to convert 6-gingerol to 6-shogaol. Second, petroleum ether—ethyl acetate (1:1) was used to increase the concentration of 6-shogaol in ginger oil extract. HPLC was conducted to estimate the amount of 6-shogaol in the ginger oil (Figure S6). The retention time of 6-shogaol in the chromatographic program was 4.194 ± 0.0059 min (RSD = 0.14%, tailing factor= 1.01, theoretical plates = 4588), and the linear regression was 166,129.76x + 22,743.83 with R^2^ = 0.9997 (Figure S6). In addition, the HPLC chromatogram of ginger oil showed a strong and clear peak at a similar position as standard shogaol (Figure 1B), confirming that this program could be used to examine the concentration of shogaol in the extracted oil. After applying ethanol extraction with heat, the amount of 6-shogaol increased from 0.22% to 0.88%. By applying *two extraction steps*, the concentration of 6-shogagol increased to 2.33% (23.33 µg per mg of ginger oil), an increase of 2.65 and 10.59 times as compared to the single solvent and the raw material, respectively. Taken together with the GC-MS results, by applying the extraction protocol described above, the volatile *compounds* comprised, by mass, 3.69% ginger oil, of which shogaol compounds constituted approximately 70%.

### 2.2. Characterization of GL Nano-Lipid

**Figure 2:**
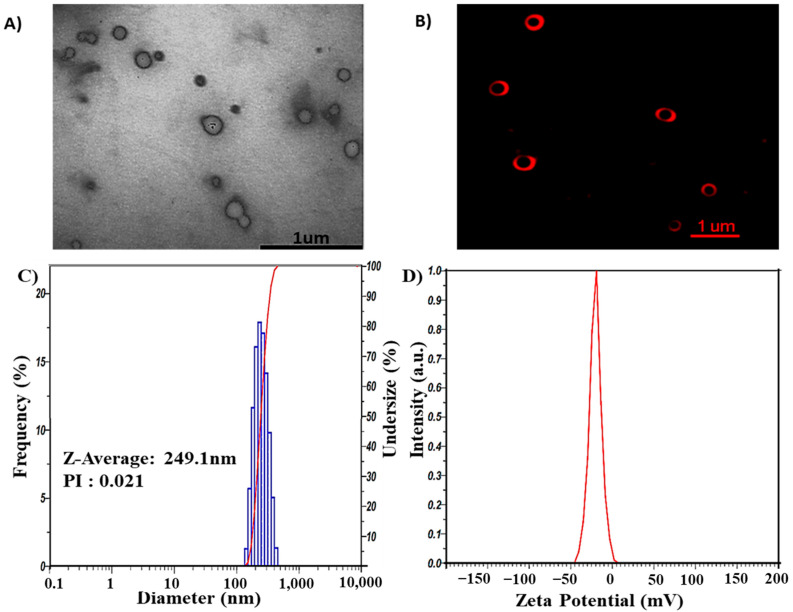
Morphology of GL nano-lipids according to TEM image (**A**) and confocal microscopy image labeled with DiL C18 (**B**). Size distribution (**C**) and ζ potential values (**D**) as obtained by DLS of GL nano-lipids at 25 °C.

GL *n*ano-lipids were fabricated through the thin-film hydration method using lecithin, cholesterol, polysorbate 80 and ginger oil. *Transmission electron microscopy (TEM)* (Figure 2A) shows that GL nano-lipid particles not subjected to the extrusion process appeared bright against the background of the heavy-metal stain, similarly to a liposome structure. However, owing to the stabilization problem of the morphology of nano-lipids in the dry stage, nano-lipids have a truncated shape. Therefore, to verify the structure of nano-lipids, the carrier was stained with Dil C18. Confocal microscopic inspection of the liquid stage showed the red fluorescence signal in the shell that emanated from the lipid bilayer (Figure 2B). GL nano-lipid morphology is similar *to a* liposome. In addition, *dynamic light scattering (DLS*) revealed that nano-lipid particles had a circular shape and a homogeneous size. The hydrodynamic *radi*us *was* 249.1 nm (Figure 2C), *and the* polydispersity index (PDI) was 0.021. These data confirm the relatively narrow size distribution of GL nano-lipids, a finding which is in agreement with the confocal imaging. The time correlation function of scattered light of GL nano-lipids showed a single exponential decay, *sug*g*esting* the high purity of GL nano-lipids *and the lack of* large particles in suspension. In addition, the participation of ginger oil in the lipid phase did not affect the electrical characteristics of lecithin–cholesterol–tween 80 colloidal nanoparticles. Soy lecithin contains a mixture of various phospholipids [11], including acidic phospholipids such as phosphatidylinositol, phosphatidylserine and phosphatidylglycerol, which confer a negative charge on the lecithin lipid droplet surface [12]. C*onventional* lecithin nano-lipids generate a negative zeta potential, about −54.5 mV [9]. After adding ginger oil, the surface charge of GL nano-lipids was less negative (Figure 2D). This finding *suggest*s that ginger oil and the lipid layer interact [28].

### 2.3. Physical Stability of GL Nano-Lipids

It is critical to evaluate the physical stability of dispersions. For lipid carriers, size as well as size distribution are crucial parameters for intravenous applications [6,7]. Unstable lipid nanoparticles could induce a fat embolism [29,30]. Thus, the hydrodynamic size and zeta potential of GL nano-lipids were *examined* during 60 days of storage.

**Figure 3:**
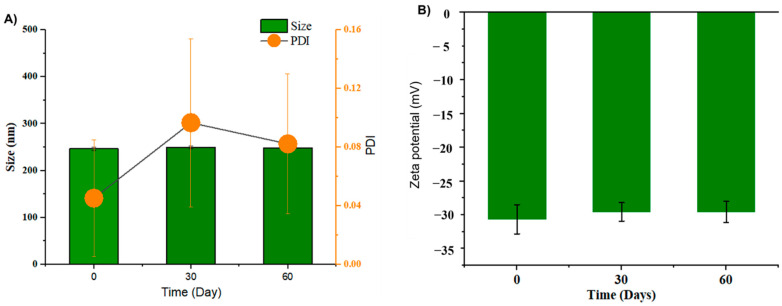
Hydrodynamic size PDI value (**A**) and zeta potential **(B)** of GL nano-lipid solution during storage time: 0 day, 30 days and 60 days at room temperature (~25 °C). Results are presented as mean  ±  standard deviation, (n  =  9).

As shown in Figure 3A, GL nano-lipids were stable with respect to the particle size distribution over 60 days of storage (*p* > 0.05). Neither the intensity nor the number of particle size distributions revealed the occurrence of a second population, which would indicate particle aggregation. In general, samples stored at 25 °C showed a noticeable increase (*p* < 0.05) in the PDI after 30 days of storage, although the value remained under 0.1, suggesting the monodistribution of nanoparticles [14]. The zeta potential varied between −30.4 mV and −29.0 mV (Figure 3B) during the 60-day *storage period*. The non-significant difference in zeta potential reveals that GL nano-lipid stability did not decrease during the entire storage period. Because the absolute values of the measured zeta potentials were above the theoretically appointed 30 mV limit required for stability [31], GL nano-lipids are acceptable for the preparation of stable nano-lipid formulations.

**Figure 4:**
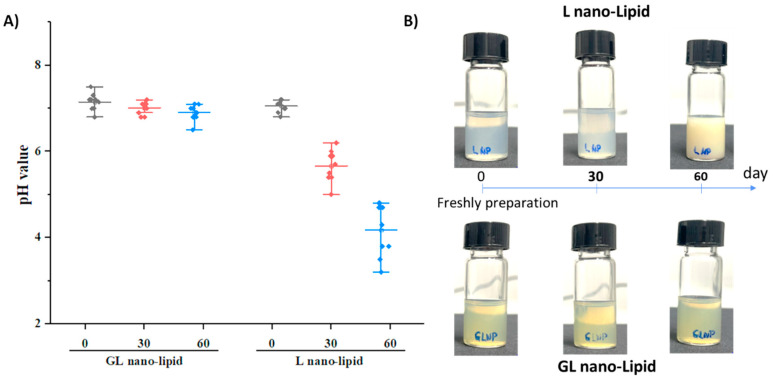
pH value (**A**) and visual observations **(B)** of GL nano-lipid and L-nano-lipid solutions during storage time: 0 day, 30 days and 60 days at 25 °C. Boxes represent pH value with a probability between 25% and 75%; the line inside the box indicates the median pH value of solution, and bullets indicate data.

A major disadvantage of nano-lipids based on soy lecithin is the critical chemical stability of the lipid layer after long*-term* storage. Lecithin is known to degrade when exposed to air, and its degradation cause*s* a decrease in pH value of suspension [32,33]. To test this phenomenon, GL nano-lipid was re-hydrated, and its pH was measured as a function of time *compared* with lecithin nano-lipids. a pH value of 7.2 was detected in both the freshly prepared GL nano-lipids and lecithin nano-lipids. However, the pH of lecithin nano-lipids decreased with time (Figure 4A, *p* > 0.05). This finding is consistent with the results obtained with lecithin liposomes [13,14]. Although the size and the PDI did *not change* after 3 months, a liposome formulation based on soy phosphatidylcholine degraded and increased the acidic level of the dispersion [14]. With the addition of ginger oil, GL nano-lipid dispersion exhibited only a very minor decrease in pH; it remained above 6.5 even after *stora*ge for 60 days. Images of lecithin nano-lipids and GL nano-lipids during storage are shown in Figure 4B. At day 60, the stored lecithin nano-lipids were turbid compared with the freshly prepared sample. However, the appearance of GL nano-lipids remained similar to that of the freshly prepared sample during the entire storage period. Along with the pH of the dispersion, the increased turbidity of lecithin nano-lipids could be due to the degradation of lecithin in *the* poorly soluble phospholipid hydrolysis product. The stable pH of GL nano-lipids might be attributed to the inclusion of ginger oil, which has various antioxidants.

A pyrogallol assay was performed to assess the contribution of ginger oil to the protection of GL nano-lipids from degradation. *P*yrogallol was oxidized by hydrogen peroxide (H_2_O_2_) with the help of horseradish peroxidase (HRP) [34]. Previous studies have mentioned the antioxidant activity of ginger oil due to the abundance of polyphenols such as 6-shogaol and gingerol [25,26]. In this assay, ginger oil or GL nano-lipids were first incubated with H_2_O_2_ before adding pyrogallol and HRP. After 30 min, an orange-brown solution and precipitate appeared in the control sample (Figure S7), indicating the formation of pyrogallol-quinone (an oxidative product of pyrogallol). However, ginger oil and GL nano-lipids did not show a color change. In other word, both ginger oil and GL nano-lipids showed scavenging activity to inhibit H_2_O_2_.

These results suggest that the inclusion of ginger oil could prevent lipid oxidation and thus enhance the stability of nano-lipids compared to the raw form.

### 2.4. Biocompatibility of GL Nano-Lipids

**Figure 5:**
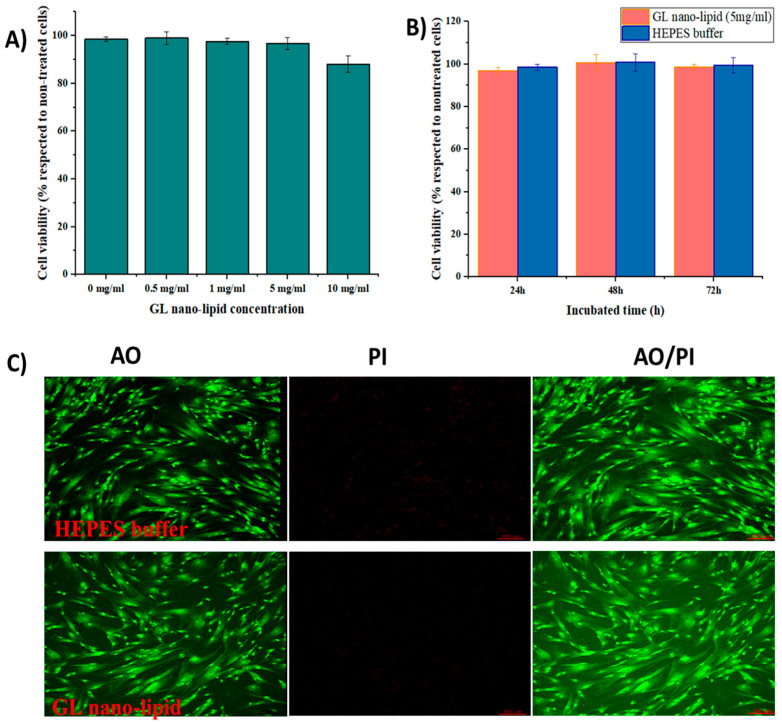
(**A**) The cytotoxicity of MSCs treated with different GL nano-lipid concentrations (0 mg/mL, 0.5 mg/mL, 1 mg/mL, 5 mg/mL and 10 mg/mL) in respected to non-treated MSCs. (**B**) The cytotoxicity of MSCs in function of time when incubated with 5 mg/mL GL nano-lipid and HEPES buffer. Results are presented as mean  ±  standard deviation (n  =  3). (**C**) Con-focal microscopy images of MSCs up to 72 h incubation at 37 °C with GL nano-lipid (5 mg/mL) and HEPES buffer. AO: green color; PI: Red color; AO/PI: Merged color; Scale bar = 150 µm.

Lecithin nano-lipids have excellent *biocompa*t*i*bi*lity* [8]; however, ginger oil *produces dose-dependent* cytotoxicity [35]. Thus, the cytotoxicity profile of GL nano-lipids was assessed with MSCs. First, MSCs were exposed to various concentrations of GL nano-lipids (Figure 5A). GL nano-lipids exhibited excellent bioavailability up to the highest concentration tested. After 24 h of exposure to 5 mg/mL GL nano-lipid, the viability of MSCs was ≥95% with respect to untreated cells. However, 10 mg/mL GL nano-lipid significantly reduced MSC growth (*p* < 0.05) by 88.07*%* ± 3.46% relative to untreated cells. Based on ISO 10993, GL nano-lipids could be classified as relatively harmless. To examine the more long-term effects, MSCs were incubated with 5 mg/mL GL nano-lipid for 72 h (Figure 5B). The MSC viability after 72 h of exposure to nanoparticles was similar to the viability after 24 h and 48 h of exposure. To evaluate the cytotoxic profile of GL nano-lipids, dual acridine orange (AO) and propidium iodide (PI) staining was conducted. As shown in Figure 5C, after 72 h incubation with GL nano-lipids, MSCs showed little change in cell survival, in agreement with the SRB result. MSCs cultured with Hepes buffer or GL nano-lipids exhibited a homogeneous fibroblastic morphology with good adhesion to the plate. After 72 h of exposure to nanoparticles, the density of the cell was indistinguishable from the negative control. These data indicate that GL nano-lipids have excellent biocompatibility, which make them good potential drug carrier candidates.

### 2.5. Antibacterial Activity of GL Nano-lipid

Ginger oil has antibacterial activity [24,25,26,36]. Hence, *Escherichia coli* and *Staphylococcus aureus* were used as model to prove the intrinsic antibacterial activity of GL nano-lipid. Ampicillin and Streptomycin served as positive controls; both exhibit strong *anti*bacterial activity (Figure S8 and Table 1). A treatment of 1.16 mg/mL ginger oil produced an inhibition zone diameter of 14.83 ± 0.58 mm *for E. coli* and 12.77 ± 1.65 mm for *S. aureus*. A treatment of 10 mg/mL lecithin nano-lipid did not produce an inhibition zone. However, 10 mg/mL GL nano-lipid produce an inhibition zone diameter of 17.20 ± 0.76 mm and 15.87 ± 0.47 mm *for E.coli* and *S. aureus*, respectively. Thus, the addition of ginger oil to lecithin nano-lipid confers antibacterial activity.

### 2.6. Use of GL Nano-Lipids to Deliver Curcumin

**Figure 6:**
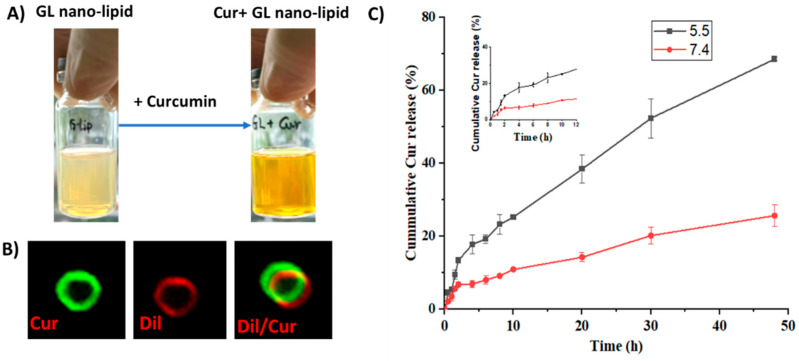
(**A**) Visual observations and confocal microscopy image (**B**) of Cur/GL nano-lipids. (**C**) In vitro release profiles of curcumin from GL nano-lipids at pH 7.4 (red line) and at pH 5.5 (black line) in 37 °C. Data are presented as mean ± SD (n = 3).

Curcumin, a bioactive compound with various pharmaceutical properties, such as anticancer, anti-inflammation and wound healing activity, was selected as a model compound to investigate whether GL nano-lipids could encapsulate drugs. Cur-loaded GL nano-lipids (Cur@GL) had a transparent *oran*g*e*-yellow *color* (Figure 6A), confirming good dispersion in the aqueous system. The entrapment efficacy (EE) and drug loading capacity (DL) are two main parameters used to evaluate the ability of a carrier [37]. *For GL nano-lipids, the curcumin entrapment efficiency was* 85.97% ± 4.03%, *and the drug loading capacity* was 16.72% ± 0.78%. The Cur@GL *particles* were about 250.2 nm (based on DLS). Curcumin has intrinsic green fluorescence, a property that allows it to be evaluated with fluorescent microscopy to confirm successful encapsulation of curcumin in GL nano-lipids [38]. Therefore, Cur@GL was examined *with con*f*ocal microscopy to confirm successful encapsulation of curcumin in GL nano-lipids*. Following incubation with Dil, Cur@GL expressed dual fluorescent channels (Figure 6B), suggesting that curcumin had been encapsulated in GL nano-lipids. In addition, the *surface charge* of Cur@GL was higher (−20.2 mV) but not significantly different from GL nano-lipids. This higher value indicates the interaction between curcumin and the lipid layer *of the Gl nano-lipid* [28] and specifies moderate stability of the colloidal system [14,31]. Interestingly, GL nano-lipids showed different release *behavior* in a biological model. In agreement with the zeta potential value, the hydrodynamic size of Cur@GL did not change after 10 days of incubation in physiology buffer, PBS 7.4 (Figure S9). In addition, after 10 days, the *entrapment efficiency of* curcumin was 84.13% ± 4.79%. This value is similar to the EE values of the freshly prepared sample (t = 0.476, *p* = 0.66 > 0.05, n = 5), suggesting that Cur@GL has high stability.

The drug release profile was another parameter used to examine *a* carrier. The curcumin release pattern of GL nano-lipids was examined at pH values of (5.5 and 7.4), which mimic the cellular *enviro*n*ment* of normal and *tumor* tissue, respectively [39]. As shown in Figure 6C, the curcumin was released faster in the acidic *medium* (pH 5.5). In the first 2 h, the curcumin release from the carrier was quite similar at both pHs. After that, curcumin was released *stably* at pH 7.4 for up to the first 10 h (Figure 6C inset). For the acidic environment, GL nano-lipids *showed* a linear release profile for curcumin. Within 50 h, ~70% of curcumin had been released at an acidic pH, which is double the amount of curcumin released at a pH of 7.4. This pH-dependent behavior of GL nano-lipids *is consistent* with a previous report [14]. In the acidic environment, soy lecithin *degradation* was accelerated [13]. The lipid layer was markedly weakened, and subsequently, more curcumin escaped from the carrier. This pH-dependent drug release profile of GL nano-lipids could reduce the toxic effects of drug on normal tissue, and could help to increase the accumulation of drugs at the *tumor* site.

Next, the release kinetics of curcumin from GL nano-lipids was examined. Five different models which are known as the suitable models for liposome/nano-lipids [40] were used fit the experimental data using KinetDS [41]. Based on R^2^, AIC, BIC and RSME values, a better fit of experimental data to the model was achieved.

As shown in Table 2 and Figure S10, neither the first-order and Higuchi models were suitable for the release data. The release data at two different pH conditions were fitted adequately with zero order, with the Korsmeyer–Peppas and Weibull models having R^2^ values higher than 0.95. However, all AIC, BIC and RSME values suggest that the release of Cur@GL at both conditions fitted to zero-order kinetics. Zero-order drug delivery systems can be useful to enhance the therapeutic values and prevent the side effect of the drug [40,42]. Due to releasing drug at a constant rate, the concentration of drug in the bloodstream could be maintained for long time; consequently, the dosing frequency was reduced [42]. The pattern release of curcumin from GL nano-lipids suggests that GL nano-lipids could be designed for sustainable delivery of hydrophobic agents (chemotherapy drug, bioactive compound). Altogether, the obtained data exhibit that GL nano-lipids can successfully encapsulate the hydrophobic drug and perform a pH-dependent release profile, indicating its potential as a carrier for drug delivery.

## 3. Materials and Methods

### 3.1. Materials

Ginger plant (Zingiber officinale) was collected in Duc Trong District, Lam Dong Province, in April 2019. The plants were identified by Dr. Nguyen Ngoc Tuan (Institute of Biotechnology and Food Technology, Industrial University of Ho Chi Minh City), and the reference specimens were stored at Institute of Applied Materials Science (IAMS). Soy Lecithin (CAS number 8002-43-5) was ordered from Tokyo Chemical Industry (TIC, Tokyo, Japan). Tween 80 (CAS number 900 5-65-6), Cholesterol (Code 110190025) came from Acros (Fairlawn, NJ, USA). Curcumin (CAS number 458-37-7) was acquired from Merck (Singapore). Cell culture reagents originated from Gibco (New York, NY, USA) and Sigma Aldrich (Singapore). All the chemical solvents in analytical study and nano-lipid fabrication were analytical-grade and purchased from Fisher Scientific (Waltham, MA, USA). All the solvents in extracted process were obtained from Chemsol (Ho Chi Minh, Vietnam).

### 3.2. Ginger Oil Preparation

#### 3.2.1. Ginger Oil Extraction

After collection and identification, the ginger root was cleaned with tap water and then dried at room temperature for 1 week. The ginger root was ground with a planetary ball mill (PM 100, RETSCH, Haan, Germany) for 30 min with 200 rpm at 25 °C. The resultant powder (50 g) was soaked in ethanol (80%, Chemsol, Viet Nam) in soxhlet extractor at 50 °C for 24 h. Vacuum filtration with Whatman filter paper (Grade No. 41) was used to collect the supernatant. The first crude extract was obtained through the rotary evaporators (N-1200A V-WD, EYELA, Tokyo, Japan). The co-solvent, petroleum ether—ethyl acetate (1:1), was applied to the ethanol crude (ratio 1 g of crude extract: 20 mL of co-solvent). The supernatant was then collected through centrifugation at 10,000 rpm, 25 °C (HERMLE, Essen, Germany). The precipitation was repeated with co-solvent 3 times. The supernatant in each extracted repetition was merged before removing solvent with rotary evaporators, resulting in the yellow oil sample. The oil sample was placed into oven vacuum (VOS-301SD, EYELA, Tokyo, Japan) at 40 °C for 1 h to further remove solvent.

#### 3.2.2. Characterization of Ginger Oil

Gas chromatography–mass spectrometry (GC-MS): The composition of the resultant ginger oil was determined with GC-MS (GC Agilent 6890 N, MS 5973 inert) equipped with HP5-MS (0.25 mm, 0.25 mm, 30 m). The sample (25 µL) was mixed with 5 mL of n-hexane and passed through a 0.45-micron filter before being injecting into the system. The injection volume was 1.0 µL. The inlet pressure by helium gas was 9.3 psi. The temperature program was as follows: initial temperature was 50 °C for 2 min, and then was increased to 80 °C at 2 °C/min, increased to 150 °C at 5 °C/min, increased to 200 °C at 10 °C /min and finally increased to 300 °C at 20 °C/min.

High-performance liquid chromatography (HPLC): HPLC (Flexar/ParkinElmer equipped with PDA) using VDSpher PUR 100 C18 column (250 × 4.6 mm, 5 µm) was conducted to qualify the amount of 6-shogaol in the obtained ginger oil. [6]-shogaol (Sigma, code 39303, lot BCBZ1777) was used for constructing the standard curve. Standard agent or sample was dissolved in methanol and filtered with 0.45-micron filter. The HPLC program, carried out for 10 min at a detecting wavelength of 230 nm, was: injection volume 20 µL, the column flow rate, 1.0 mL/min. The mobile phase was followed gradient elution with acetonitrile (A, MeCN, HPLC-grade, Fisher Scientific) and water containing 0.1% H_3_PO_4_ (B, HPLC-grade, Acros Organics) as follows: 0–3.5 min 82% A, 3.5–4.5 min 65% A, 4.5–6 min 60% A, 6–10 min 80% A.

### 3.3. Nano-Lipid Preparation

#### 3.3.1. GL Nano-Lipid Preparation

GL nano-lipid was prepared following the conventional thin-film technique. Briefly, soy lecithin (90 mg), ginger oil (5.8 mg), cholesterol (31.8 mg) and tween 80 (31.8 mg) were dissolved separately in 10 mL of chloroform (synthesis-grade, Fisher Scientific, Geel, Belgium). All the prepared solution was transferred into a 500 mL round-bottomed (RB) flask, and chloroform was added up to 50 mL. The flask was placed into sonication bath for 1–2 min to make a homogenous solution. A thin lipid film was formed in the RB flask using the rotary evaporators (N-1200A V-WD, EYELA, Tokyo, Japan). The hydration process was carried out with 10 mM HEPES buffer (code 15630080, Gibco, New York, NY, USA). After 5 h of stirring under room temperature at a high speed (1500 rpm), five freeze–thaw cycles (−80 °C–40 °C) were applied. The obtained solution was centrifuged at 12,000 rpm. The supernatant was collected and freeze-dried for further study.

#### 3.3.2. Characterization of Nano-Lipid

TEM: The GL nano-lipid solution (0.5 mg/mL) was mixed with 2% uranyl acetate. This mixture was dropped on the copper grid (Ted Pella, Inc., Redding, CA, USA) and then dried at room temperature. TEM (JEM-1400, Tokyo, Japan) was operated at 300 KV to monitor the morphology of sample.

Confocal microscopy: GL nano-lipid (0.5 mg/mL) was mixed with Dil C18 (5) (Invitrogen™, Rockford, IL, USA) at the ratio of 200:1 *w*/*w*). This solution was dropped on the slide and observed under confocal microscope (Andor, Oxford instrument, Oxford, England) at 640 nm emission.

Size, zeta potential: Size and zeta potential were monitored under Zetasizer Nano SZ (SZ-100, Horiba, Tokyo, Japan). For size measurement, 1 mg/mL GL nano-lipid was prepared. The measurement was carried out at 25 °C with general mode analysis. For zeta potential, 0.5 mg/mL GL nano-lipid was prepared and then loaded into zeta potential cell with palladium electrodes. All measurements were performed for least three independent trials.

Stability testing: GL nano-lipid (1 mg/mL) was stored in 25 mL injection vial (Sai Gon Plastic, Ho Chi Minh, Vietnam) at room temperature. At the determined time, some parameters, such as hydrodynamic size, zeta potential and pH value, of the suspension were analyzed. Each time point included 9 repetitions. The stability of GL nano-lipid under the oxidant condition was tested using pyrogallol assay. A total of 4.2 mL of GL nano-lipid (100 ppm), ginger oil (100 ppm) and HEPES buffer were incubated with 0.32 mL of H_2_O_2_ (0.02%), separately. After 20 min of incubation at room temperature, 0.64 mL of pyrogallol (50 mg/mL) was added into each vial. Then, 0.2 mL of HRP enzyme (10 mg/mL in PBS buffer) was added. After 30 min incubation and mixing, the color of solution was recorded.

### 3.4. Fabrication of Curcumin-Loaded GL Nano-Lipid

#### 3.4.1. Preparation of Cur@GL Nano-Lipid

Curcumin-loaded GL nano-lipid (Cur@GL nano-lipid) was prepared following the thin-film method. Curcumin (2%, 3.1 mg) was dissolved into chloroform and then mixed with solution containing soy lecithin, ginger oil, cholesterol and tween 80. The procedure was similar to that used for GL nano-lipid fabrication. The unloaded curcumin was removed by centrifugation (15,000 rpm in 30 min at 25 °C, Hermele Z32HK). The supernatant was collected and then freeze-dried. The yellow powder was kept at 2–8 °C for further study.

#### 3.4.2. Characterization of Cur@GL Nano-Lipid

After lyophilizing, Cur@GL nano-lipid was re-solved into DI water to perform some measurements such as size and zeta potential. The morphology of Cur@GL nano-lipid was proven under confocal microscopy with the help of Dil C18 (5).

The amount of curcumin that was encapsulated in GL nano-lipid was determined by HPLC as described in the previous study [38]. Trixton X (1 mM) was added into Cur@GL nano-lipid. The centrifugation was applied to collect curcumin. Curcumin was then re-dissolved into absolute ethanol (HPLC-grade, Fisher Scientific, Geel, Belgium). The entrapment efficiency (EE) and drug loading capacity (DL) were calculated using the following equation:

(1) $$\mathrm{EE}\;\left(\%\right)=\frac{\text{Amount of curcumin determined by HPLC}}{\text{Initial amount of curcumin}}\times 100\%$$

(2) $$\mathrm{DL}\;\left(\%\right)=\frac{\text{Amount of curcumin determined by HPLC}}{\text{Total mass of the composition in GL nano}-\text{lipid}}\times 100\%$$

The release profile of curcumin from GL nano-lipid followed the procedure of the previous study [21]. In brief, Cur@GL nano-lipid (0.5 mL) was loaded into dialysis tube 3500 kD (Spectra/Por) and then soaked into physiological buffer (pH 5.5 and pH 7.4), PBS (1X, Gibco). At the determined time, 0.5 mL of media was withdrawn and replaced with an equal volume of fresh media. The amount of curcumin released from the system was estimated using HPLC method. The release kinetic model was performed using KinetDS 2.0 [41]. The release equation with the best goodness of fit was evaluated based on the values of AIC, BIC and RSME and co-efficient of determination shown by the KinetDS result.

### 3.5. In vitro Cytotoxic Test

The toxicity of GL nano-lipid was tested with hMSC—human mesenchymal stem cells (passage number: 04, PT-2501, Lonza, Basel, Switzerland). hMSCs were seeded on 96-well plate with density of 2 × 10^4^ cells/well and cultured with completed Dulbecco’s Modified Eagle’s Medium/Nutrient Mixture F-12 Ham media (Sigma Aldrich, D8900) with 1% Penicillin-Streptomycin (Sigma Aldrich, P4333), 7.5% sodium bicarbonate (Sigma Aldrich, S5761) and 10% Fetal Bovine Serum (Sigma Aldrich, F7524). After 24 h of incubation, the culture media was withdrawn and replaced with the new culture media containing various concentrations of GL nano-lipid (0–10 mg/mL). All the cells were cultured under normal conditions: 5% CO_2_ and 90% humidity. Sulforhodamine B (SRB) assay was applied to identify the toxicity of material following the instruction of Abcam (ab235935). Further, live/dead staining via dual Acridine Orange (AO)–propidium iodide (PI) staining was conducted to obtain more evidence for the toxic profile.

### 3.6. Antibacterial Testing

*Staphylococcus aureus* (ATCC 6538) and *Escherichia coli* (ATCC8739) were selected as models for Kirby–Bauer disk diffusion susceptibility test. A total of 100 µL of inoculum (10^7^ CFU/mL) was spread on the surface agar plate using a sterilized spreader until a dry surface was obtained. Several 6 mm filter paper disks (WHA2017006, sterilized) were placed on the agar surface. A total of 20 µL of each sample (ginger oil, GL nano-lipid, convention lecithin nano-lipid, ampicillin, streptomycin and HEPES buffer) was dropped on the paper disk. The plates were cultured at 37 °C in the incubator. The diameter of the translucent zone around the paper disk was recorded after 24 h of incubation. If the diameter of the zone was greater than 6.0 mm, it was concluded to have an antibacterial property. The experiment was repeated 3 times.

### 3.7. Statistic Test

The data are presented as mean  ±  standard deviation. The graphs for GC-MS, cell cytotoxic, stability data and release profiles were made with OriginPro (2021, version 9.8, Northampton, MA, USA). For the analysis, multiple comparisons (ANOVA test) was performed together with Tukey’s multiple comparison test or least significant difference (LSD). All the statistic tests had a confidence level of 95%.

## 4. Conclusions

Despite the excellent carrier-based lecithin nano-lipid, the application of these vesicles is still hindered in drug delivery systems due to the oxidation of lecithin during storage. This study provided an alternative procedure for fabrication of nano-lipid-based lecithin. A 6-shogaol-rich oil extracted from Zingiber officinale following two solvent-phase extractions, confirmed by HPLC and GC-MS, was used as the composition in the fabricated process. The addition of ginger oil in the lipid phase with lecithin allowed the formation of functional lipid nanoparticles. After applying the film hydration technique without extrusion, the size of nanoparticles was around 250 nm, and homogenous distribution was achieved, as confirmed by DLS and confocal imaging. Through zeta potential value, it was suggested that ginger oil was blended to a lipid layer phase. Furthermore, the addition of ginger oil did not induce any significant effects on particle size distribution and zeta potential over 60 days of storage at room temperature. Notably, a fast decreasing pH value of dispersion was noted over time for lecithin nano-lipids. Soy lecithin is a mixture of phospholipids, mainly polyunsaturated fatty acids, which are easily degraded, causing the release of fatty acids into an aqueous medium, resulting in the generation of acidic pH. In our case, the pH value of GL nano-lipid dispersion was around 7.0–7.2 in the same storage conditions with lecithin nano-lipids. We assume that the introduction of ginger oil with the rich of antioxidant compounds could minimize the oxidation of soy lecithin. In addition, the modification of the conventional lecithin nano-lipids with ginger oil showed antibacterial activity against both Gram (−) and Gram (+) bacteria. Additionally, non-significant changes in viability and morphology of mesenchyme stem cells (MSCs) cultured with the GL nano-lipid as compared to the negative control revealed its excellent biocompatibility. Specially, the modified lecithin nano-lipid particles were able provide a sustained release of the curcumin—hydrophobic bioactive agent. This study presents a new strategy for developing drug-delivery-system-based soy lecithin.

## Figures and Tables

**Table 1:** Disc diffusion assay of GL nano-lipids against some pathogenic bacteria.

Test Sample	Concentration	Inhibition Zone (mm)	
		*E. coli*	*S. aureus*
Ginger oil	1.16 mg/mL	14.83 ± 0.58 ^D^	12.77 ± 1.65 ^C^
L nano-lipid	10 mg/mL	6 ^D^	6 ^D^
GL nano-lipid	10 mg/mL	17.20 ± 0.76 ^C^	15.87 ± 0.47 ^B^
Ampicillin	10µg/mL	21.19 ± 0.70 ^B^	23.89 ± 1.56 ^A^
Streptomycin	25 µg/mL	27.4 ± 0.96 ^A^	23.84 ± 0.15 ^A^
HEPES buffer		6 ^D^	6 ^D^

Values are mean ± SD of 3 independent replicates. The significant difference between variable means is presented by letters A, B, C and D following least significant difference (LSD) test, 95% confidence level.

**Table 2:** The estimated parameters, R^2^, AIC, BIC and RSME values obtained from fitting experimental release data at pH 5.5 and pH 7.4.

	Parameter	Zero Order	First Order	Higuchi	Korsmeyer–Peppas	Weibull
pH= 5.5	R^2^	0.9627	0.0894	−0.0054	0.9931	0.9931
	AIC	70.3998	153.55	110.486	72.9361	79.5854
	BIC	71.3696	154.52	111.456	73.9059	80.5552
	RSME	4.5915	146.759	24.3971	5.1033	6.7324
pH = 7.4	R^2^	0.9808	0.0899	0.0189	0.9888	0.99
	AIC	43.3975	132.69	90.6057	50.3217	53.1504
	BIC	44.3673	133.66	91.5755	51.2915	54.1202
	RSME	1.4905	61.5375	10.6559	1.989	2.2378

## Data Availability

Not applicable.

## Supplementary Materials

The following supporting information can be downloaded at: https://www.mdpi.com/article/10.3390/pharmaceutics14081654/s1, Figure S1: GC chromatograms of ginger oil; Figure S2: Mass spectrum extracted from a peak 40.41 (A) and its fragmentation (B); Figure S3: Mass spectrum extracted from a peak 41.45 (A) and its fragmentation (B); Figure S4: Mass spectrum extracted from a peak 43.88 (A) and its fragmentation (B); Figure S5: Mass spectrum extracted from a peak 23.57 (A) and its fragmentation (B); Figure S6: Standard curve of [6]-shogaol constructing by HPLC; Figure S7: The color of pyrogallol solution in the presentation of HEPES buffer, Ginger oil and GL nanolipid; Figure S8: Zones of Inhibitions formed on nutrient agar surface by ginger oil, L nano-lipid, GL nano-lipid, and two positive controls (Ampicillin and Streptomycin) against *E. coli* (A) and *S. aureus* (B).; Figure S9: Hydrodynamic size value of Cur@GL in PBS buffer and the EE values after 10 days storaged at 25 °C; Figure S10: Release kinetics of Cur@GL at pH 7.4 (A) and at pH 5.5 (B) fitted to 5 kinetic models contructing by KinetDS; Table S1: Compounds identified by GC–MS using NIST-14 Mass Spectral library.
