# Supplementary material for: Nano-Lipids Based on Ginger Oil and Lecithin as a Potential Drug Delivery System
Source: Pharmaceutics. 2022 Aug 9;14(8):1654. doi: 10.3390/pharmaceutics14081654 (PMC9412309; doi:10.3390/pharmaceutics14081654)
Supplement: Supplementary file 1 [file pharmaceutics-14-01654-s001.zip › pharmaceutics-1775453-supplementary.pdf]

Article

Nano-lipids based on ginger oil and lecithin as a potential drug delivery system

Hung Quach[1], Tuong-Vi Le[1], Thanh-Thuy Nguyen[1,2], Phuong Nguyen[3*], Cuu Khoa Nguyen[1,2*], Le Hang Dang[1,2*]

1 Institute of Applied Materials Science, Vietnam Academy of Science and Technology, HCMC, Viet Nam
2 Graduate University of Science and Technology, Vietnam Academy of Science and Technology, HCMC, Viet Nam
3 Faculty of Chemical Technology, HCMC University of Food Industry, Ho Chi Minh City, Viet Nam
* Correspondence: nckhoa@iams.vast.vn ; ntphuong@iams.vast.vn; dtlhang@iams.vast.vn

Supporting material

Table S1: Compounds identified by GC–MS using NIST-14 Mass Spectral library
Figure S1: Full GC spectrum of ginger oil.
Figure S2: Mass spectrum extracted from a peak 40.41 (A) and its fragmentation (B)
Figure S3: Mass spectrum extracted from a peak 41.45(A) and its fragmentation (B)
Figure S4: Mass spectrum extracted from a peak 43.88 (A) and its fragmentation (B)
Figure S5: Mass spectrum extracted from a peak 23.57 (A) and its fragmentation (B)
Figure S6: Standard curve of [6]-shogaol constructing by HPLC
Figure S7: The color of pyrogallol solution in the presentation of HEPES buffer, Ginger oil and GL nano-lipid
Figure S8: Zones of Inhibitions formed on nutrient agar surface by ginger oil, L nano-lipid, GL nano-lipid, and two positive controls (Ampicillin and Streptomycin) against E.*coli* (A) and S.*aureus* (B).
Figure S9: Hydrodynamic size value of Cur@ GL in PBS buffer and the EE values after 10 days storaged at 25°C.
Figure S10: Release kinetics of Cur@ GL at pH 7.4 (A) and at pH 5.5 (B) fitted to 5 kinetic models contructing by KinetDS

Table S1: Compounds identified by GC–MS using NIST-14 Mass Spectral library

Peak	R.T.	Name of compound	Pct Total
1	23.584	Geraniol	1.949
2	37.032	n.i	0.474
3	39.479	n.i	1.378
4	40.138	n.i	3.113
5	40.41	(6)-Shogaol	63.137
6	41.549	[8]-Shogaol	6.29
7	41.644	n.i	5.09
8	42.449	n.i	1.438
9	42.679	n.i	5.671
10	43.871	Geranyl oleate	3.735
11	45.544	[6]-Gingerdiol (2E)-geranial acetal	7.726

Figure list

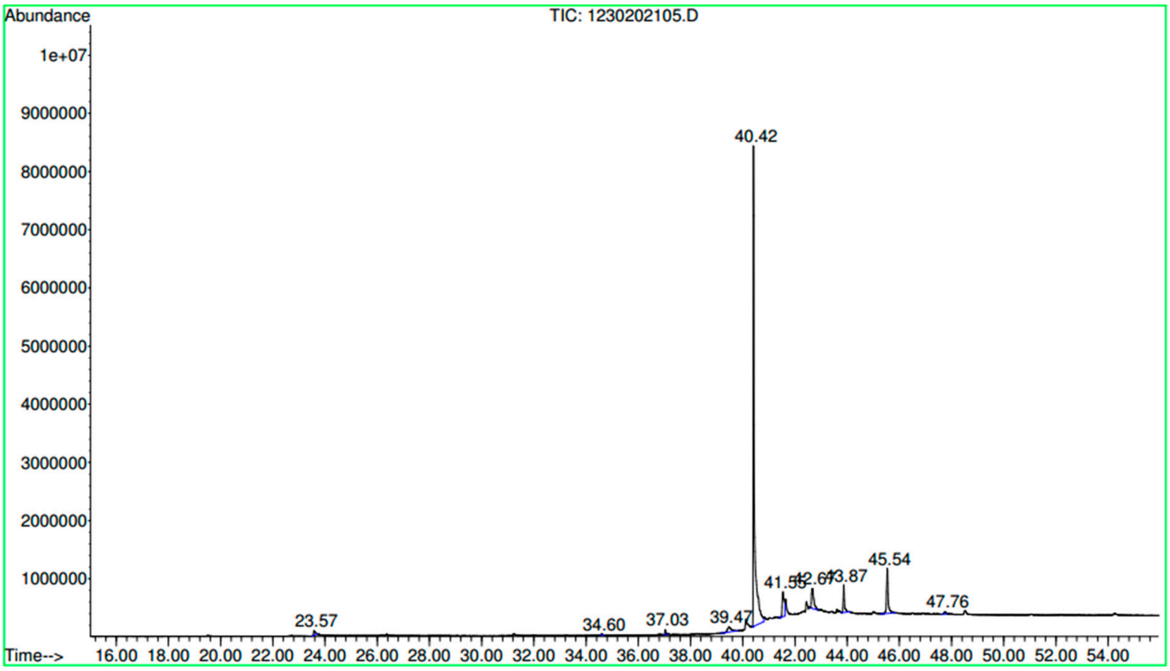

Figure S1: Full GC spectrum of ginger oil

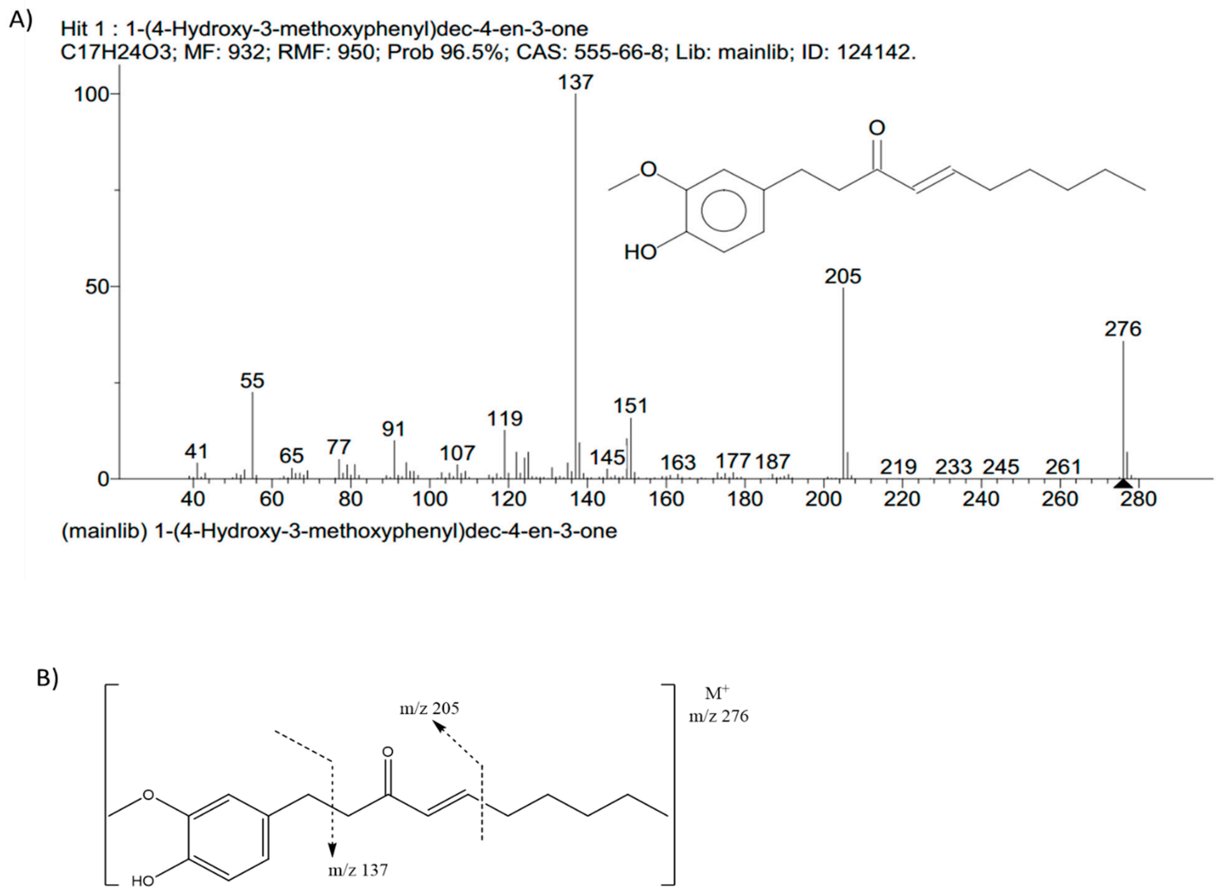

Figure S2: Mass spectrum extracted from a peak 40.41 (A) and its fragmentation (B)

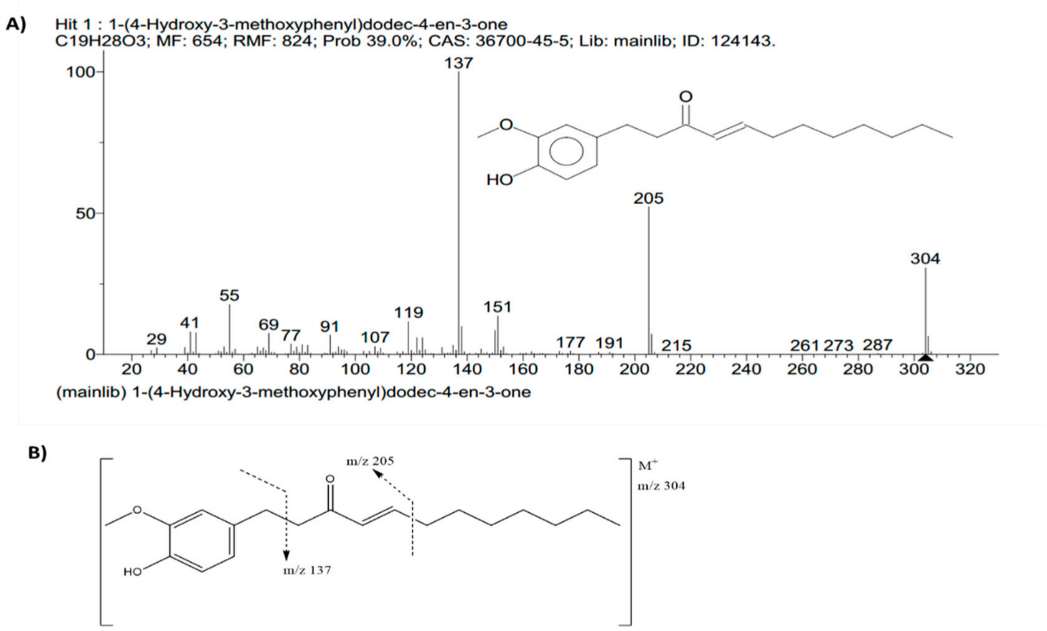

Figure S3: Mass spectrum extracted from a peak 41.45(A) and its fragmentation (B)

A)

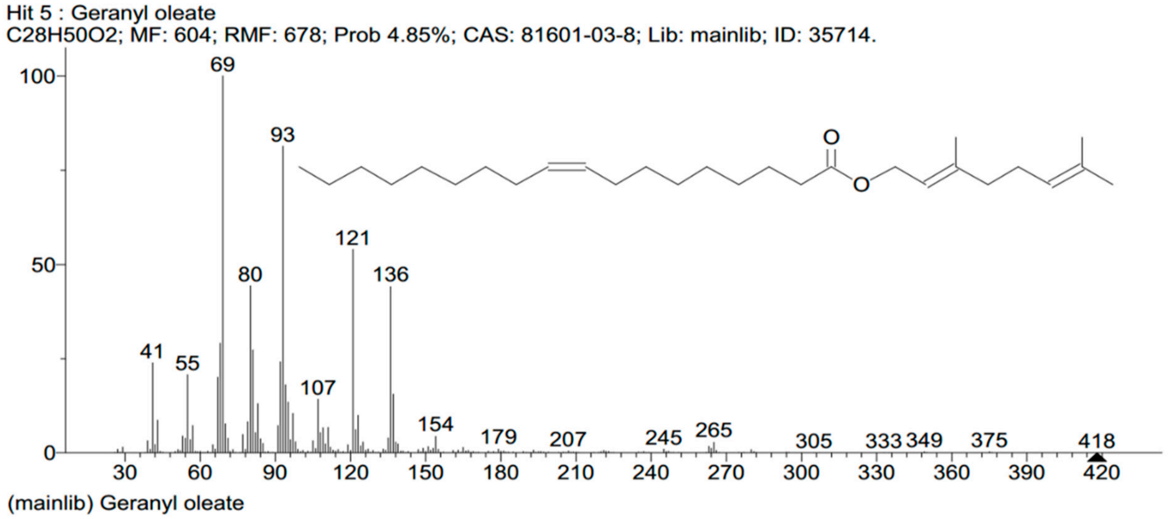

B)

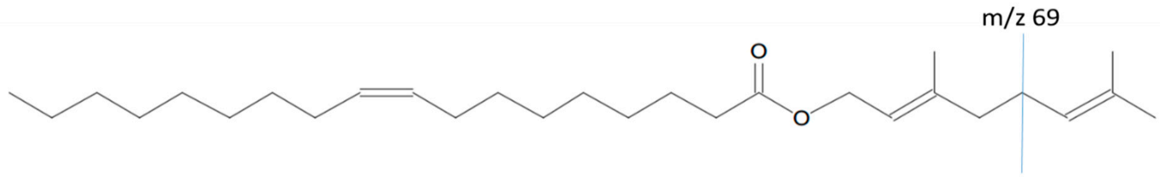

Figure S4: Mass spectrum extracted from a peak 43.88 (A) and its fragmentation (B)

A)

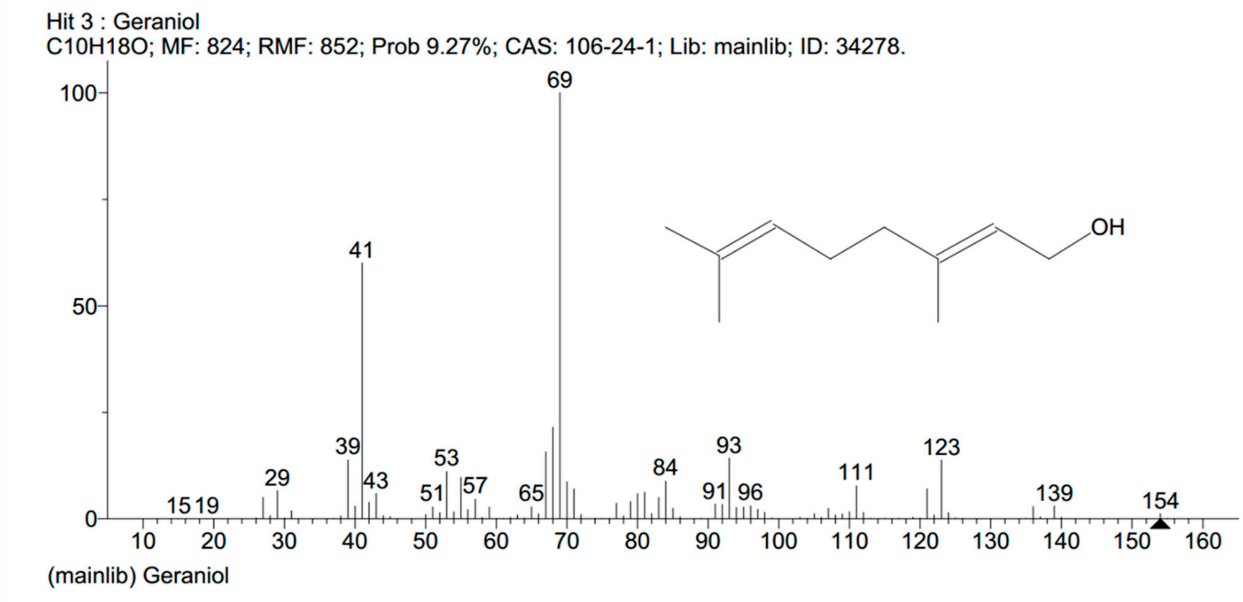

B)

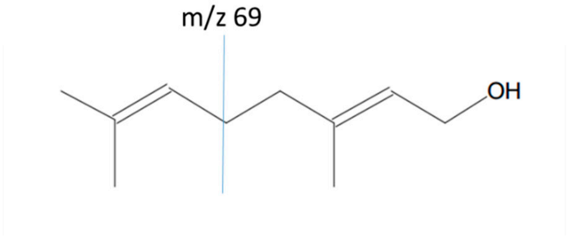

Figure S5: Mass spectrum extracted from a peak 23.57 (A) and its fragmentation (B)

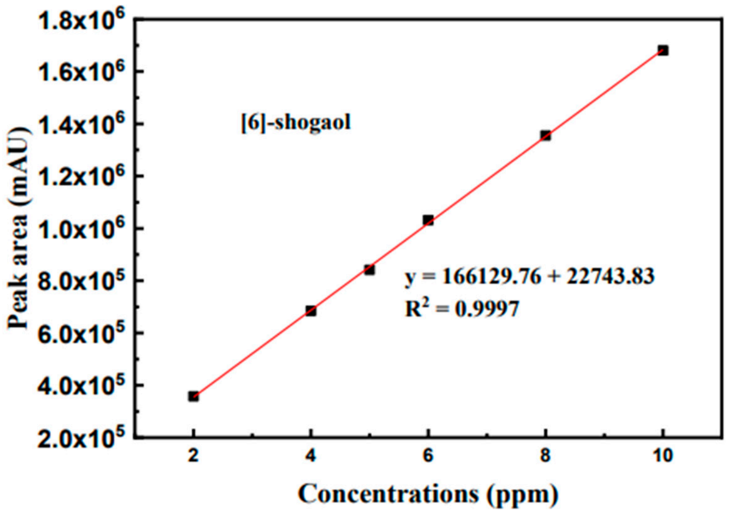

Figure S6: Standard curve of [6]-shogaol constructing by HPLC

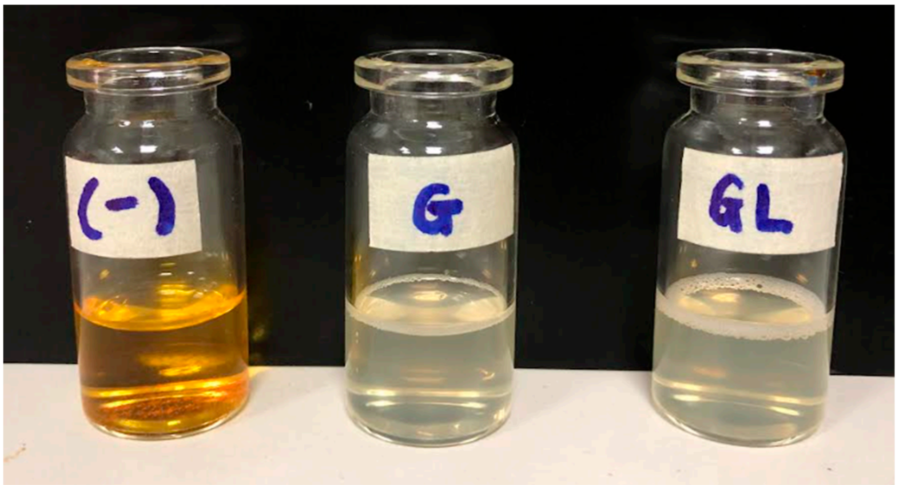

Figure S7: The color of pyrogallol solution in the presentation of HEPES buffer, Ginger oil and GL nano-lipid

A) B)

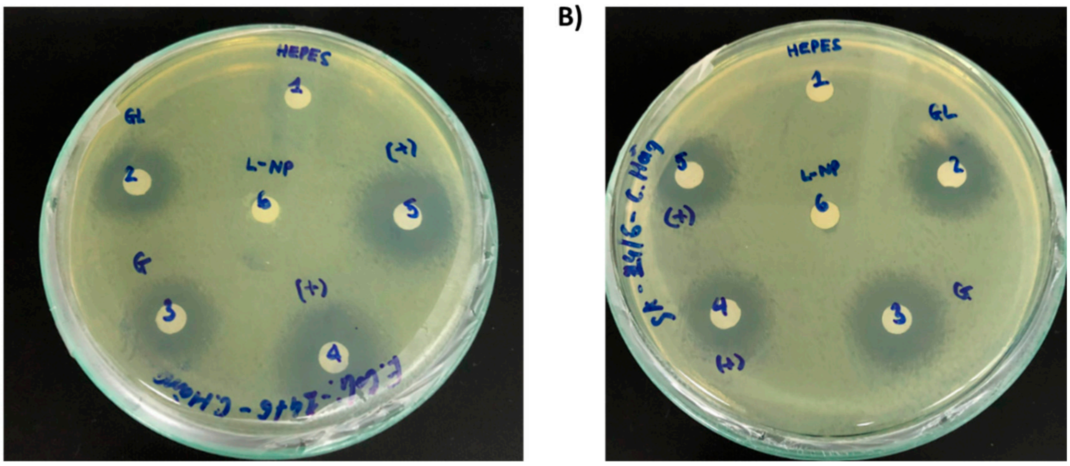

Figure S8: Zones of Inhibitions formed on nutrient agar surface by Hepes (1) GL nano-lipid (2), ginger oil (3), and two positive controls (Ampicillin (4) and Streptomycin(5)) L nano-lipid (6) against E.*coli* (A) and S.*aureus* (B).

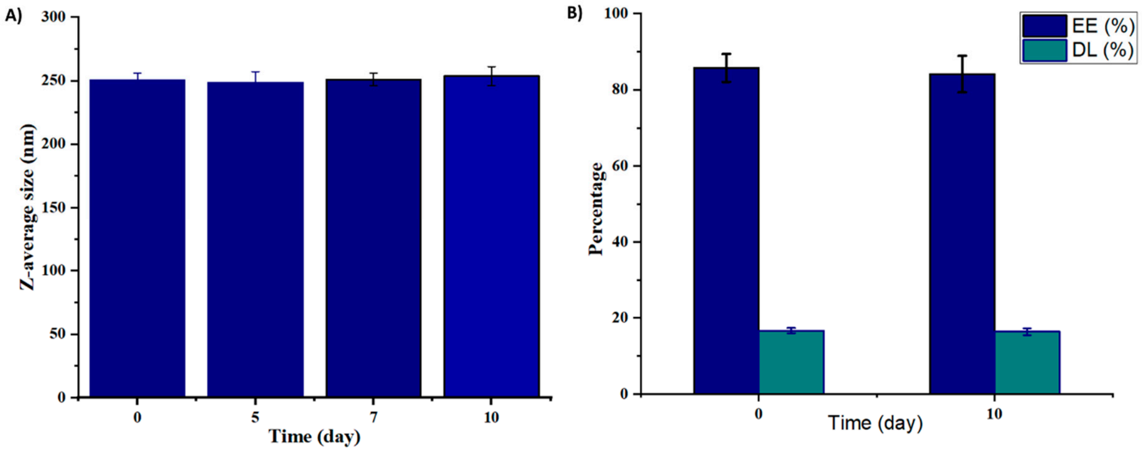

Figure S9: Hydrodynamic size value of Cur@ GL in PBS buffer (A) and the EE and DL values (B) after 10 days storaged at 25°C.

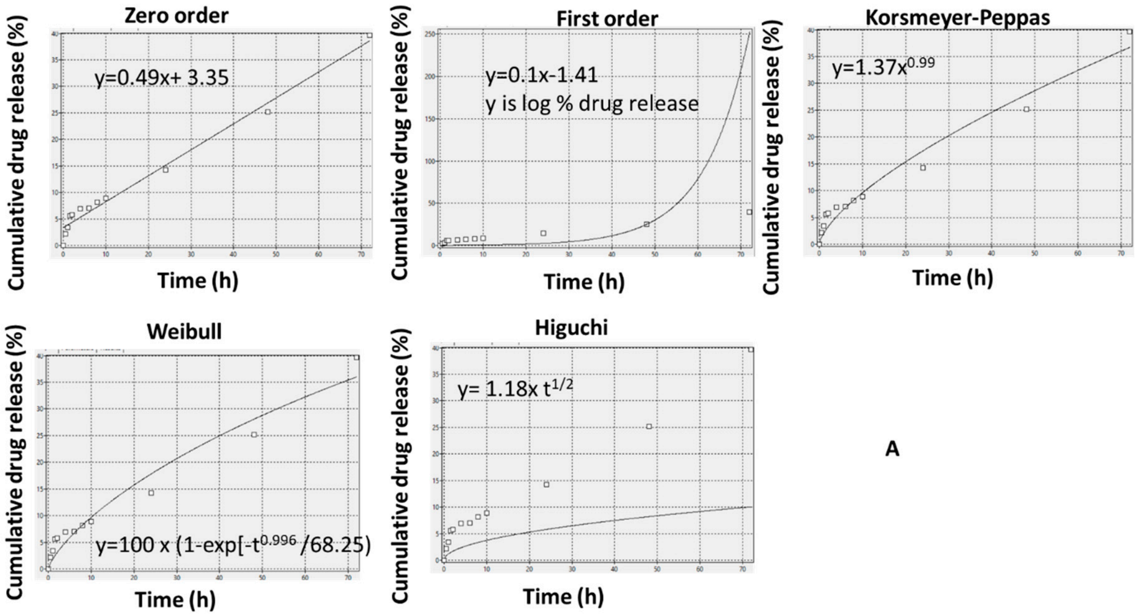

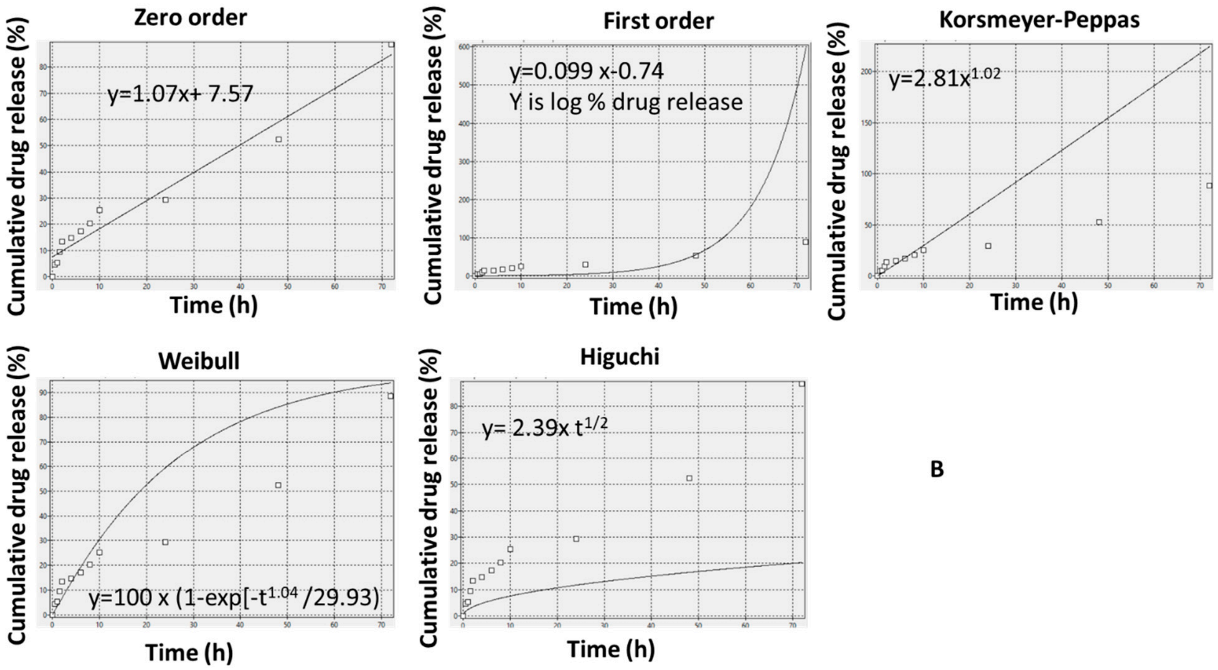

Figure S10: Release kinetics of Cur@ GL at pH 7.4 (A) and at pH 5.5 (B) fitted to 5 kinetic models contructing by KinetDS
